# Adherence to Accelerated Diagnostic Protocol for Chest Pain in Five Emergency Departments in Canada

**DOI:** 10.5811/westjem.48701

**Published:** 2025-12-31

**Authors:** Jesse Hill, Esther Yang, Shandra Doran, Michelle M. Graham, Sean van Diepen, Joshua E. Raizman, Albert KY Tsui, Brian H. Rowe

**Affiliations:** * University of Alberta, College of Health Sciences, Faculty of Medicine and Dentistry, Department of Emergency Medicine, Edmonton, Alberta, Canada; † Alberta Health Services, The Alberta Strategy for Patient-Oriented Research Support Unit, Edmonton, Alberta, Canada; ‡ University of Alberta, College of Health Sciences, Faculty of Medicine and Dentistry, Department of Medicine, Division of Cardiology, Mazankowski Heart Institute, Edmonton, Alberta, Canada; § University of Alberta, College of Health Sciences, Faculty of Medicine and Dentistry, Department of Laboratory Medicine and Pathology, Edmonton, Alberta, Canada; || Alberta Precision Laboratories, Edmonton, Alberta, Canada; # University of Alberta, College of Health Sciences, School of Public Health, Edmonton, Alberta, Canada

## Abstract

**Introduction:**

In this study we sought to to assess the extent to which emergency physicians adhered to an institutional protocol for rapid chest pain assessment that incorporates a high sensitivity troponin I (hs-TnI) assay. We also sought to characterize clinical outcomes stratified by protocol adherence.

**Methods:**

We conducted a retrospective cohort study that included all adult patients presenting to five major metropolitan hospital emergency departments (ED) with suspected cardiac chest pain who had at least one troponin measured. The study period was November 9, 2020–June 20, 2022. The primary outcome was protocol adherence for indeterminate-risk and high-risk patients, as defined by the protocol in use at the time of each patient’s presentation to hospital. Adjusted odds ratios (aOR) are reported with associated 95% confidence intervals.

**Results:**

A total of 14,027 patients were included in the study, among whom 8,962 (63.9%) were classified as low risk, 4,064 (29.0%) as indeterminate risk, and 1,001 (7.1%) who were in the high-risk/rule-in group. Overall, 35.9% of patients had care that adhered to the chest pain pathway protocol—22.1% of indeterminate-risk patients and 91.6% of high-risk/rule-in patients. Protocol adherence among indeterminate-risk patients was 6.6% when the initial troponin was in the range of 4–19 nanograms per liter (ng/L) and 75.4% for initial troponin levels 20–99 ng/L. Male sex was most strongly associated with protocol adherence; among those receiving adherent care, 65.8% were male compared to 34.2% female (aOR 1.67; 95% CI, 1.46–1.91). Patients in the non-adherent group with an initial troponin 4–19 ng/L experienced a significantly higher incidence of major adverse cardiac events (4.5% vs 1.7%, *P* < .001), compared to those in the low-risk group.

**Conclusion:**

Adherence to proposed assessment protocols for patients presenting to the ED with chest pain was low. This lack of adherence appears to disproportionally affect females and is associated with poor outcomes. Improving adherence to evidence-based guidelines in this setting is urgently needed.

## INTRODUCTION

Protocolized care for common conditions is increasingly prevalent within the emergency department (ED) setting. Despite their evidence-based development and implementation,^1^–^3^ little is known regarding the extent to which clinicians are compliant/adherent to the protocols. Widespread use of electronic health record systems within EDs facilitates more comprehensive assessments of adherence to these protocols.^4^

Within the realm of acute coronary syndromes, adherence to certain protocols within the ED has previously been documented to be low. Protocol adherence may reflect the timing of troponin draws, the number of repeat troponin tests required, when to seek cardiology consultation, and when to arrange outpatient stress testing.^5^ Accelerated protocols have become mainstream since the widespread adoption of high-sensitivity troponins.^1^,^2^ These protocols have been shown to decrease ED length of stay, without a corresponding increase in 30-day major adverse cardiac events (MACE) and, in many cases, reducing MACE.^1^

Non-adherence to these protocols poses a potential safety risk. One of the more common initial accelerated diagnostic pathways was the HEART Pathway.^6^ Secondary analysis of the primary research demonstrated that among the relatively small sample size of 141 patients, 20% were not investigated as outlined in the protocol.^7^ Fortunately, they did not identify any increase in MACE among these patients; however, the small sample size limited the detection of these rare events.

Our urban health system with several tertiary-care hospitals EDs, in collaboration with cardiology and laboratory medicine, hs implemented accelerated diagnostic protocols associated with high-sensitivity troponin (hs-Tn) assays. All sites are staffed 24 hours/day almost exclusively by Royal College or Family Medicine-Emergency Medicine graduates and receive a variety of learners throughout the year. These changes represent an ideal opportunity to study physician adherence to this important diagnostic protocol as well as the possible changes in patient outcomes based on protocol-compliant care. Furthermore, the relative recency of establishing new protocols may provide insight into whether recurrent protocol changes accelerate waning compliance. Our goal in this study was to explore protocol adherence to an institutional diagnostic protocol for the workup of chest pain in the ED, with a secondary focus on major outcomes (eg, consultation, admission, and adverse events) after the introduction of the protocol.

## METHODS

### Ethics

The study was approved by the University of Albert Health Research Ethics Board (HREB) at the University of Alberta, in Edmonton, Alberta, Canada (reference ID: Pro00145421). Approval was also obtained to access electronic health records from several administrative databases. Through a waiver of informed consent provided by the HREB, we did not obtain written informed consent from any patients whose data were included in the study. Operational and administrative approvals were provided by Alberta Health Services (AHS), and a data-sharing agreement was signed. The physicians practicing during the study period were unaware of any studies at the time of data collection.

**Population Health Research Capsule**

What do we already know about this issue?

*Accelerated protocols for assessment of chest pain have become mainstream in emergency departments; they are safe and improve efficiency*.

What was the research question?

*We sought to assess the extent of physician adherence to rapid chest pain assessment protocols*.

What was the major finding of the study?

*Overall, 35.9% of patients had care that adhered to the chest pain pathway protocol*.

How does this improve population health?

*Patients receiving non-adherent care with a single troponin measurement have worse outcomes. Further research into strategies to improve compliance is warranted*.

### Design

We conducted a retrospective cohort study of all adult patients (> 17 years of age) presenting to the five major EDs in the Edmonton area (University of Alberta Hospital, Sturgeon Hospital, Misericordia Hospital, Grey Nuns Hospital, and Royal Alexandra Hospital, minimum annual ED census > 48,000) with chest pain of cardiac origin who had at least one troponin ordered during their ED visit. Chest pain of cardiac origin is defined as a Canadian Triage and Acuity Scale (CTAS) score of II (emergent) and excludes chest pain patients assessed as CTAS I (Resuscitation) and CTAS III (urgent, although assessed as non-cardiac).

The study period was November 9, 2020–June 20, 2022. This corresponds to the introduction of a chest pain assessment protocol using a hs-TnI assay (Beckman Coulter Diagnostics, Brea, CA) and three-hour protocol across the five EDs.^2^ The Beckman hs-TnI assay demonstrated excellent precision and achieved a coefficient of variation of ≤ 10% at the overall 99^th^ upper reference limit of 18 nanograms per liter (ng/L) across all five sites. The limit of detection was established at 3 ng/L. There were significant educational efforts made in advance of the protocol implementation. A brief video was created and distributed together with a “Survival Guide” to emergency physicians and internal medicine and cardiology physicians, zone wide. A paper-based version of the protocol was distributed to all participating EDs. Emergency clinicians received an in-service on use of the protocol and had the opportunity to ask questions or clarify details. Finally, immediately prior to implementation, a laboratory bulletin was sent through Medical Affairs’ secure e-mail channels to remind staff of the upcoming changes.

### Protocol

Results are sub-classified based on cumulative or serial (“delta”) troponin results that map out to the three risk categories of the chest pain protocols (see Appendix A). Negative/low-risk patients require the following:

- Single hs-TnI test value ≤ 3 ng/L and symptoms > 3 hours; *OR*
- Serial hs-TnI tests: first and second test value <20 ng/L and delta (0–3 hours) ≤ 5 ng/L.

To be classified as indeterminate risk, patients require the following laboratory criteria:

- Single hs-TnI test value between 4–99 ng/L; *OR*
- Serial hs-TnI tests:
  ○ First test value < 20 ng/L and delta (0–3 hours) 5–25 ng/L; OR
  ○ First test value 20–99 ng/L and delta < 5 ng/L; OR
  ○ First test value 20–99 ng/L and delta 5–25 ng/L.

Similarly, to be considered high-risk, the following criteria must be met:

- Any single hs-TnI value ≥ 100 ng/L; *OR*
- Serial troponin tests:
  ○ First test value 20–99 ng/L and delta > 25 ng/L.

Details regarding the timing of any individual patient’s chest pain onset were not available in this retrospective, administrative data study. Given that the indeterminate- and high-risk groups always require further ED management they represent ideal targets for assessment of protocol adherence. The following patients were excluded from the analysis: those with ST-segment elevation myocardial infarction, those who left without being seen by a qualified physician or nurse practitioner, and patients without at least one high-sensitivity troponin measurement. Additionally, non-residents of Alberta were excluded due to lack of available post-ED outcome data. In the event of multiple visits, we included the data from the initial visit for each patient.

### Data Sources

We accessed province-wide administrative databases to obtain retrospective data. All the datasets accessed are maintained and updated in the AHS Enterprise Data Warehouse and are linked using unique patient identifiers. Specifically, the following were used: National Ambulatory Care Reporting System, which captures all ED visits across provinces using *International Classification of Disease and Related Health Problems, 10^th^ Revision, Canada* [*ICD-10-CA*] diagnostic codes and patient basic demographics); the Emergency Department Information Tracking System captures ED visits in Edmonton and possesses information regarding patients clinical and demographic characteristics; Connect Care and AHS Lab datasets provide information about the laboratory investigations performed during the ED visits; provincial diagnostic imaging contains records about the imaging investigations (eg, chest radiographs, computed tomography) performed during ED visits; Provincial Registry and Alberta Vital Statistics capture death within 30 days of ED discharge; Practitioners Claims (captures all physician billing claims and includes several visit diagnoses); and Discharge Abstract Data, which captures all hospital admissions and interventions. We observed optimal chart review practices: data extractors were trained; data and variables were clearly defined; databases were defined as above; sampling methods were clear; and institutional and ethics approval was obtained.^8^

### Outcomes

Our primary outcome was protocol adherence, defined as follows:

- No serial troponin measurement for the indeterminate group; *OR*
- First hs-TnI value > 99 ng/L with no cardiology consult or hospitalisation; *OR*
- Serial hs-TnI delta > 25 ng/L, with no cardiology consult or hospitalisation.

Secondary outcomes included any differences in demographics and clinical outcomes (30-day MACE, stroke, myocardial infarction [MI], death, and cardiac interventions) based on protocol adherence. Further, we also sought to evaluate protocol adherence as a function of 1) time since protocol implementation and 2) magnitude of initial troponin result.

Given the significant disparity in protocol adherence between high-risk and indeterminate-risk groups, we elected to focus specifically on the outcomes within the indeterminate-risk group to avoid unintentionally highlighting differences between the risk groups, rather than differences between protocol adherence and non-adherence.

### Statistical Analysis

We calculated descriptive statistics for all included patients. Baseline differences between the chest pain pathway classification and comorbidities are reported. We reported data using means and standard deviations, medians and interquartile ranges or proportions with 95% confidence intervals, as appropriate. Comparisons were made using the Student *t*-test for continuous parametric data and Wilcoxon test for continuous non-parametric data; dichotomous data was compared using a chi-squared test. We used a multivariable logistic regression to explore the factors associated with adherence. Primary outcome results were considered statistically significant at a 2-tailed *P*-value < .05. For all other tests, significance was set at *P* < .001.

## RESULTS

### Demographics

A total of 14,027 patients met criteria for inclusion. Of these patients, 8,962 (63.9%) met hs-TnI result criteria for the negative/low-risk group. There were 4,064 (29.0%) and 1,001 (7.1%) patients in the indeterminate- and high-risk/rule-in groups, respectively. Median age was 53 (IQR 40–65) in the negative/low-risk group compared to 65 (IQR 52–76) amongst indeterminate patients. Indeterminate- or high-risk patients were also more likely to arrive by ambulance. Similar trends are observed across a range of relevant pre-existing conditions.

Table 1 illustrates the demographic differences between the protocol adherent and non-adherent patient groups in the indeterminate- and high-risk cohorts. The median age for protocol-adherent patients was older (68 vs 63 years of age; *P* < .001). Male sex was more common in the adherent group (65.8% vs 55.4%, *P* < .001). Ambulance arrival was significantly higher in the adherent group (54.7% vs 35.9%, *P* < .001). A history of hypertension, coronary artery disease, diabetes, and diabetes mellitus were all more common among patients for whom the protocol was followed.

### Protocol Adherence

Overall, 35.9% of patients experienced protocol-adherent care (Table 2). The proportions varied between the two subgroups with 22.1% of indeterminate-risk patients and 91.6% of high-risk/rule-in patients receiving protocol-adherent care. Of the 84 high-risk patients who did not experience protocol adherence, 54 were due to an elevated initial troponin without corresponding consultation/ hospitalization. Among indeterminate-risk patients receiving non-adherent care, only 9.4% had a recent ED visit with a recorded troponin value. Adherence over time is shown in the Figure. There was a trend towards decreased compliance over time (*P* = .002).

In the indeterminate-risk group, we divided initial troponin results into increments of 10 to assess whether there were obvious cutoffs for adherence (Table 3). Adherence when the initial troponin was 4–9 nanograms per liter (ng/L), or 10–19 ng/L was 2% and 21%, respectively, and rose to 78% when the troponin was 20–29 ng/L. We conducted a multivariable logistic regression analysis exploring variables associated with protocol adherence among indeterminate- and high-risk patients. Variables included time since protocol introduction (per six months), sex, age (in five-year increments), Charlson Comorbidity Index score, and initial troponin result (per 10 ng/L). After adjustment, male sex had the strongest association with protocol adherence (adjusted odds ratio [aOR] 1.67; 95% CI, 1.46–1.91). Age (aOR 1.11; 95% CI, 1.09–1.14) and initial troponin result (aOR 1.05; 95% CI, 1.04–1.06) were weakly associated with adherence. There was no difference between adherence across the five sites (*P* = .002).

### Outcomes

There were significant differences in the outcomes between patients who received protocol-adherent care and those who did not (Table 4). More patients experiencing protocol-adherent care were admitted to hospital (33.8 vs 19.0%, *P* < .001). The time to initial physician assessment was significantly lower amongst patients receiving protocol-adherent care, 69 (37, 124) minutes, compared to 89 (45, 157) minutes (median difference −20 minutes; 95% CI, −25.7 to −14.3). The overall median ED length of stay was reduced by 226 minutes (95% CI, −250.6 to −201.4) amongst patients who did not receive protocol-adherent care. Patients in the adherent-care category experienced significantly higher proportions of MI, cardiac interventions, death, and MACE.

We compared the outcomes of patients in the low-risk group to those of patients who received non-adherent care but were potentially low risk if they had an appropriate serial troponin (NA-LST [non-adherent, low single troponin] (ie, single troponin 4–19 ng/L). Patients in the NA-LST group experienced significantly higher MACE (4.5% vs 1.7%, *P* < .001; Table 5). Admissions (14.2% vs 5.0%, *P* <.001) and ED consultations (17.2% vs. 10.7%, *P* <.001) were also higher in the NA-LST group.

## DISCUSSION

In a large, urban, multicenter analysis of patients presenting to the ED with suspected cardiac chest pain, we evaluated adherence and outcomes associated with a novel institutional chest pain assessment pathway employing hs-TnI. We note the following: Overall adherence to the protocol was extremely low; protocol adherence appeared poorest for patients with a low initial troponin as well as amongst those lacking conventional cardiac risk factors; and while non-adherence is associated with many factors, the strongest association was seen in female patients. Finally, patient outcomes were worse among patients with a low initial troponin receiving non-adherent care. Using robust and linked administrative data, this evaluation provides a detailed evaluation of chest patient assessment in five EDs in one Canadian region.

Using a large sample over two years, we collected patients at high risk for cardiovascular diseases based on risk factors. Despite this, nearly two in three patients were classified as presenting with negative/low-risk chest pain. A larger proportion of older and male patients were assigned to the indeterminate- and high-risk groups, and more patients had higher comorbidity index scoring. While it makes intuitive sense that patients with higher troponin results are more likely to be older and have more baseline comorbidities, other factors contributed to these findings.

Despite extensive efforts to educate clinicians and reporting the hs-TnI results with the protocol recommendations in the EHR,^2^,^3^ clinicians’ overall adherence to the protocol was low. There were observed variations between severity sub-groups. Adherence appeared to decline after implementation (a so-called “decay”). Adherence decay is not limited to the ED. This phenomenon has been observed in outpatient settings as well (eg, rheumatologists treating patients with rheumatoid arthritis exhibited significantly lower protocol adherence three years after a protocol was rolled out^9^). We examined adherence at individual hospital sites and found similar non-compliance across all sites. Moreover, compliance with patients in the high-risk category was higher than those in the indeterminate-risk category. Educational strategies such as meetings or distributed educational materials have been shown to improve protocol adherence.^4^ Perhaps distributing these at more regular intervals (ie, annually) rather than solely during the rollout of the protocol could help combat decay.

There are a few possible explanations for adherence decay. Adherence among clinicians managing high-risk patients was generally good, approaching 92%. This may indicate that cardiologists being consulted support these hs-Tn upper-limit cutpoints and emergency clinicians face fewer barriers to patient transfer, or that emergency clinicians are less comfortable deviating from evidence-based protocols in patients who they feel may have a higher risk of poor outcomes. Conversely, indeterminate-risk patients were much less likely to receive per-protocol adherent care. Patients in the indeterminate-risk category received non-adherent care when initial troponin were elevated but below 99 ng/L. As outlined in the protocol, these patients should have received a second hs-TnI measurement.

Subgroup analysis of patients by their initial troponin value revealed a clear distinction between clinician management of patients with initial troponins, which theoretically could have placed the patient into the low-risk/rule-out group (NA-LST). Overall protocol adherence with an initial troponin < 20 ng/L was only 6.6%. It is possible that these patients would have had a troponin measurement that would have led them to be classified in the low-risk group; however, without the second measure, the true classification is impossible to know. Any troponin level above this threshold (hs-TnI > 20) had adherence rates approaching 70–80%. While this level of adherence is more in keeping with previously published literature,^7^ it does not reflect on the protocol or the expectation of the developers.

There are several possible explanations for this low compliance: either clinicians are misinterpreting the protocol, or they feel comfortable with a single troponin measurement in many of these patients. It is also possible that some of the behavior is driven by concerns for expedient discharges/efficiency in the face of ED crowding, either intentionally or subconsciously. Anecdotally, many patients have chronic mild elevations in their high-sensitivity troponins that in our system can be reviewed easily on the EHR. Many physicians are comfortable with a single troponin if it is in keeping with the patient’s known mild chronic elevation. We sought to quantify this by examining the number of patients who had a recent ED visit (within six months) and a prior troponin test (Table 2). Only 9.4% of patients who received non-adherent care had a recent troponin level to reference (and only 70% of these patients had an elevated result), suggesting that chronic troponin elevations are not a major driver of protocol non-compliance.

Treating these NA-LST patients the same as low-risk patients is unsafe. The risk of MACE for NA-LST was more than double (2.65x) compared to patients with protocol-adherent, low-risk care, presumably due to reclassification into the indeterminate-risk group. The fact that NA-LST patients had more consults and admissions does suggest that clinicians appreciate some risk difference, even in the face of protocol non-adherence.

Initial troponin results were not strongly correlated with protocol adherence and seemed to be less important to protocol adherence than the passing of the upper margin of a potential low-risk troponin value (hs-TnI, 20). Male sex was the variable most strongly associated with protocol adherence (aOR 1.67; 95% CI, 1.46–1.91)). Patients identified as male more commonly received protocol-adherent care, which is in keeping with previous research showing that patients identified as female are significantly less likely to receive serial troponin measurements, or evidence-based treatments, when presenting to the ED with chest pain.^10^,^11^

Table 1 illustrates the demographic differences between the two patient cohorts. Patients who received protocol-adherent care were more likely to have several traditional cardiac risk factors. They were more likely to be male, older, arrive by ambulance, have a history of hypertension, coronary artery disease, diabetes, and heart failure. Clinicians use a multifactorial approach when assessing a patient presenting with chest pain; while protocols driven by lab values are important, clinicians seem likely to be more cautious with a patient who has higher risk based on their past medical history than someone who is young and lacking in conventional risk factors. Correspondingly, clinical outcomes between the indeterminate-risk patients receiving non-adherent care vs adherent care (Table 4) seem to reflect a difference in risk profiles.

Shorter ED LOS among patients receiving non-adherent care almost certainly reflects early discharge rather than improved care. This is a potentially dangerous finding in a setting of increased pressure to combat ED crowding and drive down LOS; physicians with a higher proportion of non-adherent care may have “better” LOS metrics. Patients who received protocol-adherent care were 78% more likely to be admitted to hospital. The proportion of patients experiencing an MI, or MACE within 30 days, was over three times higher among indeterminate-risk patients receiving protocol-adherent care. While there is limited evidence to suggest the protocol-adherent care is leading to worse clinical outcomes, it is far more likely that these patients represent higher baseline-risk individuals.

## LIMITATIONS

There are several important limitations to this study. First, all included sites operate within a single city in the Canadian healthcare system; this system provides healthcare services to all registered citizens without direct charge. Second, we obtained data from administrative databases, which are limited by their retrospective nature, and by what data were collected contemporaneously by clinicians. Important behavioral risk factors such as smoking, obesity, alcohol use, exercise, and diet are not well captured in these databases.

Third, by excluding CTAS III patients it is probable we overlooked many cases of cardiac chest pain; however, this was considered acceptable given the multitude of non-cardiac related issues triaged as a CTAS III chest pain that may appropriately be worked up in a non-protocolized fashion. Details regarding the timing of any individual patient’s chest pain onset were not available in this retrospective, administrative-data study, which limited our ability to accurately assess which patients were managed according to protocol in the negative/low-risk group. It is possible that patients received non-protocol indicated additional troponin measurements (ie, a repeat troponin for a patient with an initial troponin undetectable with pain present >3 hours) but were unable to discern this.

Fourth, the designated subgroups were based exclusively on troponin results. The nature of chest pain (typical, atypical) or its duration (hours vs days), the electrocardiogram (ECG) results (eg, normal, non-specific changes, dynamic ST changes, flipped or hyperacute T-waves, etc) or historical factors used in other accelerated diagnostic protocols (ADPs) (eg, HEAR or HEART) were not considered.^7^ Abnormal ECG appearances may prompt appropriate deviations in care from our protocol and could have been falsely regarded as non-adherent care in our study. Finally, protocol non-adherence may be driven by a multitude of factors (ie, clinical/administrative/etc), which cannot be assessed retrospectively.

## CONCLUSION

Compliance with multidisciplinary, evidence-based, and widely disseminated protocols for patients presenting to the ED with chest pain in this healthcare system was low, especially for patients with initial low-level hs-TnI measures. Approximately one in three patients presenting to the ED with chest pain received protocol-adherent care from emergency physicians. They were more likely to follow the protocol in patients who have traditional risk factors for coronary artery disease, which has implications for patients who may present with atypical symptoms and fewer risk factors, especially women.^11^ Patients receiving non-adherent care with a single troponin measurement did have worse outcomes. Clearly, patients with a minimally elevated initial troponin who did not have a repeat level sent are receiving sub-optimal and potentially dangerous care. Further research into strategies to improve compliance is warranted. Changes such as automatic EHR notifications, regular educational updates, or reflex-ordering may be effective.

## Supplementary Information



## Figures and Tables

**Figure:**
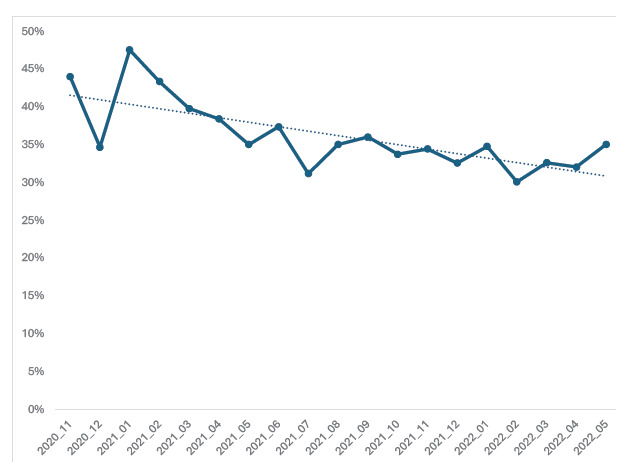
Protocol adherence for patients presenting to emergency departments with chest pain by month after the introduction of an accelerated chest pain assessment protocol at five Canadian emergency departments.

**Table 1:** Demographic characteristics between patients presenting to five Canadian emergency departments with chest pain receiving protocol adherent or non-adherent care.

Patient demographics	Total N = 5,065	Non-adherent n = 3,249	Adherent n = 1,816	P-value
Age (years), Median (IQR)	65 (53, 76)	63 (50, 74)	68 (57, 80)	< .001
Male sex (n [%])	2,993 (59.1)	1,799 (55.4)	1,194 (65.8)	< .001
Mode of arrival (n [%])				
EMS	2,161 (42.7)	1,167 (35.9)	994 (54.7)	< .001
Time of day (n {%})	5.064	3,249	1,815	< .001
Daytime (8:01 am – 4 pm)	2,223 (43.9)	1,466 (45.1)	757 (41.7)	
Evening (4 pm–12 am)	1,940 (38.3)	1,263 (38.9)	677 (37.3)	
Early morning (1 am – 8 am)	901 (17.8)	520 (16.0)	381 (21.0)	
Pre-existing conditions (n [%])				
Hypertension	2,638 (52.1)	1,591 (49.0)	1,047 (57.7)	< .001
CAD	1,727 (34.1)	803 (24.7)	924 (50.9)	< .001
Diabetes mellitus	1,411 (27.9)	806 (24.8)	605 (33.3)	< .001
Atrial fibrillation	1,111 (21.9)	628 (19.3)	483 (26.6)	< .001
Stroke	691 (13.6)	390 (12.0)	301 (16.6)	< .001
Asthma	285 (5.6)	196 (6.0)	89 (4.9)	.09
Heart failure	718 (14.2)	320 (9.9)	398 (21.9)	< .001
COPD	494 (9.8)	293 (9.0)	201 (11.1)	.02
Charlson Comorbidity Index Score				
Median (IQR)	1 (0, 2)	1 (0, 2)	1 (0, 3)	<0.0001

Adherent and non-adherent groups include both indeterminate- and high-risk patients.

*CAD*, coronary artery disease; *COPD*, chronic obstructive pulmonary disease; *EMS*, emergency medical services.

**Table 2:** Proportion of indeterminate- and high-risk patients experiencing protocol-adherent care amongst patients presenting to five Canadian emergency departments with cardiac chest pain during the study period.

	Total 5,065	Indeterminate 4,064	High Risk 1,001
Protocol adherence			
Yes, n (%)	1,816 (35.9)	899 (22.1)	917 (91.6)
ED visits within the prior 6 months and received a troponin test	215 (11.8)	129 (14.4)	86 (9.4)
No, n (%)	3,249 (64.2)	3,165 (77.9)	84 (8.4)
ED visits within the prior 6 months and received a troponin test	312 (9.6)	297 (9.4)	15 (17.9)
Non-Adherent Category			
No second troponin measurement for the indeterminate group	3,165	3,165	NA
First hs-TnI value > 99 ng/L, with no consult or hospitalization	54	NA	54
Serial hs-TnI delta > 25 ng/L, with no consult or hospitalization	39	NA	39

*ED*, emergency department; *hs-Tnl*, high-sensitivity troponin l, *ng/L*, nanograms per liter.

**Table 3:** Protocol adherence for indeterminate-risk patients presenting to five Canadian emergency departments with cardiac chest pain during the study period, based on initial troponin measurement.

hs-TnI (ng/L) Initial	Total	Non-adherent	Adherent	Adherent %
4–9	2,496	2,422	74	3%
10–19	610	479	131	21%
20–29	482	106	376	78%
30–39	255	70	185	73%
40–49	148	36	112	76%
50–59	93	21	72	77%
60–69	76	17	59	78%
70–79	57	18	39	68%
80–89	49	18	31	63%
90–99	35	8	27	77%
	4,301	3,195	1,106	

*hs-TnI*, high-sensitivity troponin I assay; *ng/L*, nanograms per liter.

**Table 4:** Outcomes for patients presenting to emergency departments with cardiac chest pain, stratified to indeterminate risk based on protocol-adherent vs non-adherent care after the implementation of accelerated pathways using a high-sensitivity troponin assay at five Canadian emergency departments.

N	Total 4,064	Non-adherent 3,165	Adherent 899	P-value	median differences (95% CI)
Disposition (n {%})				**< .001**	
Admitted	904 (22.2)	600 (19.0)	304 (33.8)		
Discharged	2,975 (73.2)	2,424 (76.6)	551 (61.3)		
Transferred	123 (3.0)	86 (2.7)	37 (4.1)		
LAMA	62 (1.5)	55 (1.7)	7 (0.8)		
Emergency physician initial assessment time	4051	3153	898		
(median {IQR})	84 (42, 151)	89 (45, 157)	69 (37, 124)		20.0 (14.3 to 25.7)
ED length of stay (median [IQR])					
Overall	415 (299, 611)	372 (272, 525)	598 (455, 899)		−226.0 (−250.6 to −201.4)
Discharged	375 (276, 498)	343 (258, 456.5)	506 (412, 646)		−163.0 (−181.0 to −145.0)
Chest imaging (n [%])					
Chest radiograph	3,523 (86.7)	2,697 (85.2)	826 (92.9)	**< .001**	
Chest CT	461 (11.3)	331 (10.5)	130 (14.5)	**< .001**	
V/Q	50 (1.2)	24 (0.8)	26 (2.9)	**< .001**	
ED consultation (n [%])					
Yes	1,088 (26.8)	662 (20.9)	426 (47.4)	**< .001**	
Clinical outcomes within 30 days (n [%])					
Stroke	20 (0.5)	12 (0.4)	8 (0.9)	.05	
MI	126 (3.1)	62 (2.0)	64 (7.1)	**< .001**	
Cardiac interventions‡	169 (4.2)	102 (3.2)	67 (7.5)	**< .001**	
Death	81 (2.0)	46 (1.5)	35 (3.9)	**<.001**	
MACE*	443 (10.9)	234 (7.4)	209 (23.3)	**< .001**	

Values are n (%) or median (IQR). Boldface on values indicates a statistically significant result.

‡ Cardiac interventions include coronary artery bypass graft surgery and percutaneous coronary intervention.

* MACE is defined as a composite of all-cause death, hospitalization for heart failure, hospitalization or/and ED visit for stroke or MI, or cardiac interventions.

*ED*, emergency department; *LAMA*, leaving against medical advice; *MI*, myocardial infarction; *MACE*, major adverse cardiac event; *V/Q*, ventilation/perfusion.

**Table 5:** Outcomes among patients presenting to emergency departments with cardiac chest pain and confirmed negative/low-risk stratification, compared to those with a single troponin 4–19 ng/L (i.e. non-adherent but potentially low risk).

N	Total 11,855	Negative 8,962	Single troponin 4–19 ng/L 2,893	P-value	median differences
Disposition (n {%})				**< .001**	
Admitted	862 (7.3)	452 (5.0)	410 (14.2)		
Discharged	10,677 (90.1)	8,320 (92.8)	2,357 (81.5)		
Transferred	210 (1.8)	135 (1.5)	75 (2.6)		
LAMA	105 (0.9)	54 (0.6)	51 (1.8)		
Died	1 (0.01)	1 (0.01)	0		
Emergency clinician initial assessment	11,822	8,939	2,883		
(median [IQR])	86 (45, 154)	84 (44, 151)	95 (49, 163)		−11.0 (−15.5 to −6.5)
ED length of stay (median [IQR])					
Overall	376 (280, 501)	379 (284, 497)	367 (269, 516)		12.0 (3.9 to 20.1)
Discharged					
Chest imaging (n [%])					
Chest radiograph	10,558 (89.1)	8,072 (90.1)	2,486 (85.9)	**< .001**	
Chest CT	1,191 (10.1)	882 (9.8)	309 (10.7)	.19	
V/Q	122 (1.0)	98 (1.1)	24 (0.8)	.22	
ED Consultation (n [%])					
Yes	1,453 (12.3)	955 (10.7)	498 (17.2)	**< .001**	
Clinical outcomes within 30 days (n [%])					
Stroke	23 (0.2)	13 (0.2)	10 (0.4)	.03	
MI	34 (0.3)	24 (0.3)	10 (0.4)	.50	
Cardiac interventions‡	134 (1.1)	81 (0.9)	53 (1.8)	**< .001**	
Death	46 (0.4)	17 (0.2)	29 (1.0)	**< .001**	
MACE*	285 (2.4)	155 (1.7)	130 (4.5)	**< .001**	

Values are n (%) or median (IQR). Boldface on values indicates a statistically significant result.

‡ Cardiac interventions include coronary artery bypass graft surgery and percutaneous coronary intervention.

* MACE is defined as a composite of all-cause death, hospitalization for heart failure, hospitalization or/and ED visit for stroke or MI, or cardiac interventions.

*CT*, computed tomography*; ED*, emergency department; *LAMA*, leaving against medical advice; *MI*, myocardial infarction;; *MACE*, major adverse cardiac event; *V/Q*, ventilation/perfusion.
