# Supplementary material for: Adherence to Accelerated Diagnostic Protocol for Chest Pain in Five Emergency Departments in Canada
Source: West J Emerg Med. 2025 Dec 31;27(1):205–13. doi: 10.5811/westjem.48701 (PMC12815536; doi:10.5811/westjem.48701)
Supplement: Supplementary file 1 [file wjem-27-205-s001.pdf]

2. Edmonton rapid chest pain protocol

The below schematic outlines the Edmonton rapid chest pain protocol using the Beckman hs-TnI assay.

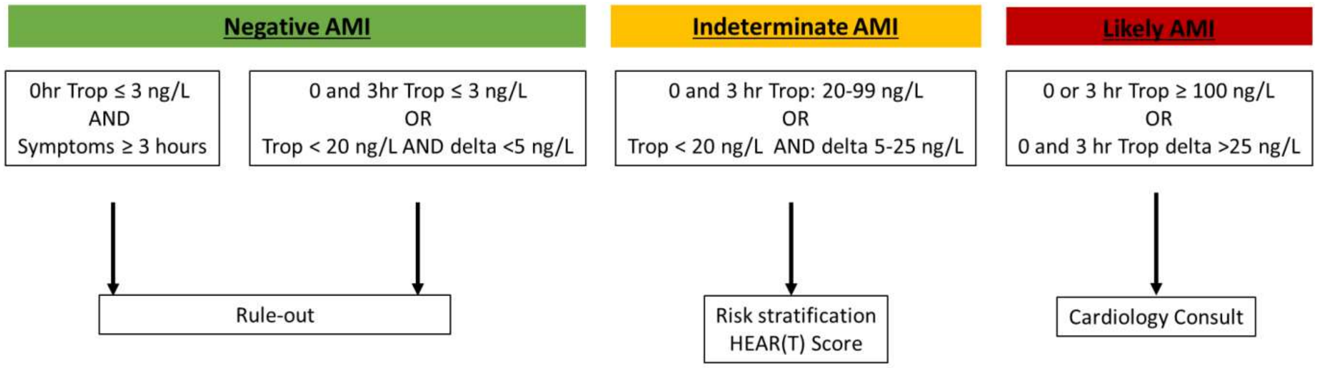

- Assuming biomarkers are collected at least 3 hours following the start of chest pain/symptoms suggestive of ACS:
    - Levels of ≤ 3 ng/L are considered safe for rule-out with a single test.
    - Levels of ≥ 100 ng/L are considered safe for rule-in;
    - Levels of 4-99 are indeterminate and need additional hs-TnI testing in 3 hours to determine the delta (or change) in hs-TnI:
        - Delta < 5 ng/L are safe for rule-out;
        - Delta 5 – 25 ng/L require risk stratification using HEAR(T) score;
        - Delta > 25 are considered rule-in.
- **Negative AMI** patients (expected ~70% of cases) – follow-up with regular primary care provider.
- **Likely AMI** patients (expected ~10% of cases) – consult Cardiology.
- **Indeterminate AMI** category (expected ~20% of cases) – risk stratify:
    - Low Risk HEAR(T) Score – outpatient referral for EST/maximize medical management;
    - Moderate Risk HEAR(T) Score – suggested outpatient referral for ***urgent*** *stress testing*;
    - High-Risk HEAR(T) Score – consultation with Cardiologist.
- HEART SCORE[7]
    - Clinical decision rule allowing rapid risk stratification of undifferentiated chest pain patients, according to their short-term risk for a Major Adverse Cardiac Event (MACE).
    - Cumulative score 0 – 10 of increasing risk for MACE.
    - MACE defined as all-cause mortality, myocardial infarction, or coronary revascularization within 30 days.
    - HEAR(T) categories (MACE Risk):
        - 0-3 = low risk (1-2%);
